# Factors Controlling Methane in Arctic Lakes of Southwest Greenland

**DOI:** 10.1371/journal.pone.0159642

**Published:** 2016-07-25

**Authors:** Robert M. Northington, Jasmine E. Saros

**Affiliations:** Climate Change Institute, University of Maine, Orono, ME, 04469, United States of America; University of Aveiro, PORTUGAL

## Abstract

We surveyed 15 lakes during the growing season of 2014 in Arctic lakes of southwest Greenland to determine which factors influence methane concentrations in these systems. Methane averaged 2.5 μmol L^-1^ in lakes, but varied a great deal across the landscape with lakes on older landscapes farther from the ice sheet margin having some of the highest values of methane reported in lakes in the northern hemisphere (125 μmol L^-1^). The most important factors influencing methane in Greenland lakes included ionic composition (SO_4_, Na, Cl) and chlorophyll *a* in the water column. DOC concentrations were also related to methane, but the short length of the study likely underestimated the influence and timing of DOC on methane concentrations in the region. Atmospheric methane concentrations are increasing globally, with freshwater ecosystems in northern latitudes continuing to serve as potentially large sources in the future. Much less is known about how freshwater lakes in Greenland fit in the global methane budget compared to other, more well-studied areas of the Arctic, hence our work provides essential data for a more complete view of this rapidly changing region.

## Introduction

Arctic regions are experiencing some of the most drastic, abrupt changes in climate compared with many other parts of the world. Temperatures in northern latitudes have increased by an average of 2–3°C since the mid 1980’s [1], and areas around Greenland are experiencing increases of 3–5°C and a doubling in the length of summer growing seasons over the past 30 years [2]. Concurrent with these changes are increases in the release of important greenhouse gases, such as CO_2_ and especially CH_4_ [1]. Atmospheric methane has shown significant increases since 2007 [3], with 16–20 Tg CH_4_ yr^-1^ greater emissions globally compared to earlier in the decade [4]. The enhanced warming of Arctic regions [2] likely contributed to substantial increases in aquatic-derived methane in the region since 2007[4]. Increasing levels of methane around the Arctic are especially concerning given the strong radiative forcing of this gas [4] and positive feedback to warming [5].

Aquatic ecosystems are some of the most important sources of methane to the atmosphere [6], especially in the Arctic [4, 7, 8]. Enhanced contemporary aquatic production of methane has been strongly linked to the landscape due, in part, to release of previously stored carbon (C) from permafrost landscapes and export from terrestrial to aquatic systems [9]. In turn, this organic material provides resources for methanogenic microbes that, in anoxic conditions, produce methane through a variety of pathways [10–12]. As methane moves from the sediments into an aerobic water column, it may be rapidly oxidized [10, 13–15], leading to a decrease in methane closer to the surface of lakes [16–18].

Understanding of the variety of factors affecting methane production in Arctic ecosystems is necessary given that surface water methane emissions have been increasing across northern latitudes in recent decades [3, 6, 7]. For example, sulfate-reducing microbes often compete with methanogens for resources, generally leading to an inverse relationship between methane production and sulfate availability in aquatic systems [19, 20]. Even so, this is not always the case, as sulfate reducers can coexist with methanogens, with each process continuing with minor competition between microbial groups [21, 22]. Additionally, recent evidence from the Alaskan arctic has suggested that methanogenesis in the active layer of permafrost soils leads to flushing of methane into lakes during melt periods [23], which may contribute further methane to the water column outside of that which is derived from sediments alone. Previous work in lakes of the Canadian Arctic [24] has demonstrated the production of methane in oxic conditions, which was strongly linked to water column primary production. Clearly, methane availability can be regulated by a variety of factors besides the availability of basal resources due solely to permafrost thaw.

Much of the current knowledge of methane dynamics in the Arctic come from sediment flux measurements [11, 25, 26], ebullition estimates [5, 8, 27], or remote sensing [28, 29]. These cross-arctic studies reinforce the importance of understanding methane across multiple scales, and provide the context within which to better understand the controls of methane within lake systems. In spite of this, much of these data focus on more well-studied Arctic regions such as Alaska [11, 15, 25, 30, 31] and Siberia [5, 8, 9, 27]. However, Greenland is one of the most rapidly warming parts of the Arctic [2, 32], with ice-free regions of the island receiving much less attention as important contributors to global change. Recent evidence of sub-glacial methane cycling and release from the Greenland Ice Sheet [33–35] suggests that the landscapes in this region will greatly contribute to future methane fluxes in the Arctic. Even so, much less is known about the role of ice-free areas of Greenland in the global methane budget. Although recent studies have suggested that Greenlandic terrestrial ecosystems serve as large sinks for methane [36], the >20,000 lakes across the ice-free landscape [37] could serve as sources for methane release to the atmosphere [6, 7].

To better understand the factors affecting methane within lakes, we surveyed 15 lakes across the ice-free regions of southwest Greenland during summer 2014. These lakes lie in a variable landscape of shrub and grass tundra and exposed rock along a gradient of temperature and age from the ice sheet outward toward the coast underlain by continuous permafrost [38]. We developed a regression model to determine the strongest predictors of lake methane concentrations, and verified the model using data collected in 2013 from 12 lakes in the region.

## Materials and Methods

### Site description

Southwest Greenland represents the largest extent of ice-free landscape in Greenland [39], with a low arctic, continental climate with average summer temperatures of 10.2°C and precipitation of 172 mm y^-1^. The summer ice-free period lasts from May/June to September. The active layer of permafrost approaches 1m in some areas [40]. The landscape varies in age with distance from the ice sheet [39], with younger, more recently exposed areas closer to the ice sheet edge. Tundra vegetation varies across the landscape, but is dominated by graminoids (*Poa pratensis*) and deciduous shrubs (*Betula nana* and *Salix glauca*) [40, 41]. Shrub extent has increased in recent years [41] and many areas are influenced by aeolian dust deposition from sandurs in the nearby Watson River [42, 43]. Surface water connections between lakes are rare [37, 38] with hydrology mostly driven by deep-permafrost ice wedges and limited precipitation during the summer [44].

**Fig 1:**
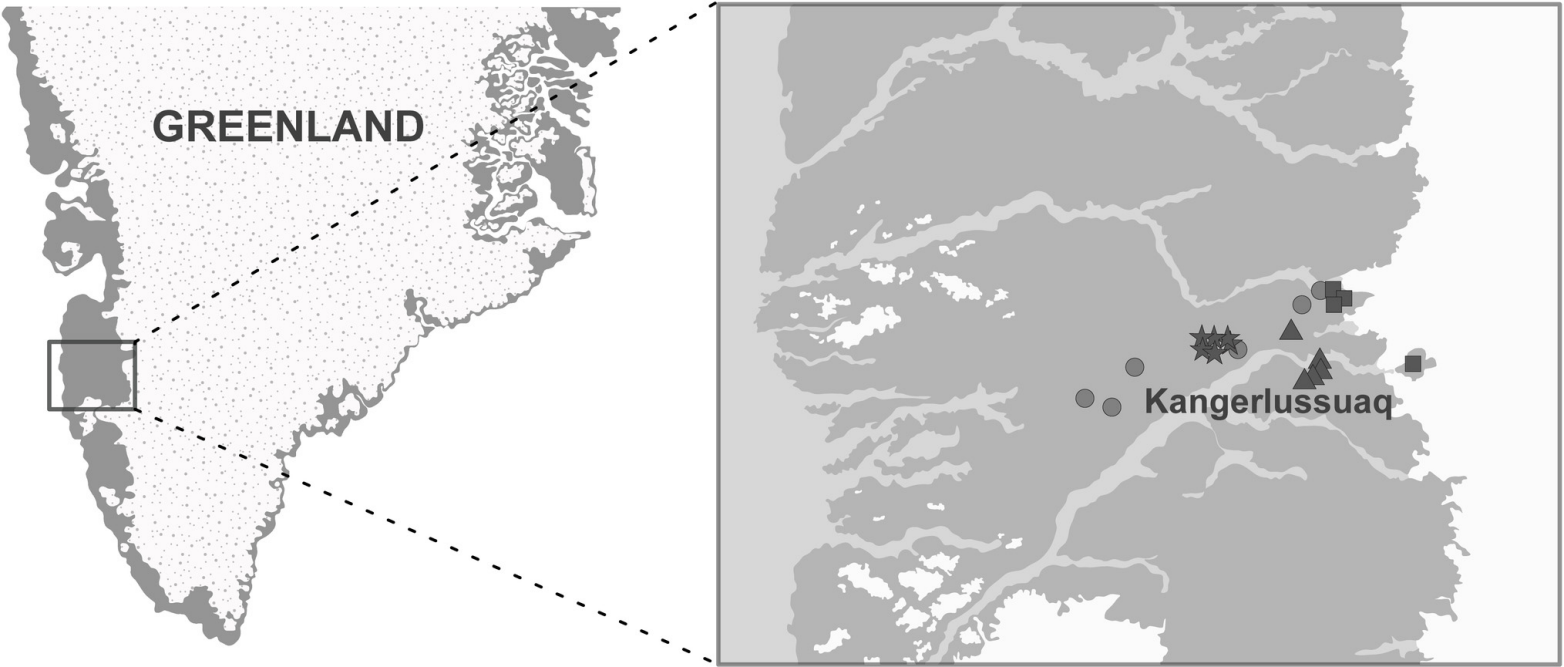
Distribution of the 15 study lakes in southwest Greenland along a 50-km southwesterly transect from the ice sheet. Lakes were classified into clusters for analysis (see Table 1) as indicated by the following symbols: Cluster A = squares, Cluster B = triangles, Cluster C = stars, lakes sampled in 2013 = circles.

During the ice-free season of 2014 (June to August), we sampled 15 lakes in the vicinity of Kangerlussuaq, Greenland along a roughly 50-km southwesterly transect from the ice sheet edge (Fig 1, Table 1). This region of Greenland is part of the Kangerlussuaq International Science Support (KISS) system that serves an international hub of research and logistical support throughout the area. As such, our field study took place with the support of the local community and no special permission was needed to access our lakes. Our work did not involve any endangered or protected species, only lakewater analyses (see methods below). Lakes were categorized into clusters based on distance from the ice sheet, with lakes in Cluster A ranging from a nunatak within the ice sheet (SS32) to lakes <10 km from its edge. Cluster B lakes were 18–20 km from the ice sheet, and Cluster C lakes were >35 km. Our earlier sampling dates occurred between 14 and 28 June, within 10 days of ice-out on all lakes. Late-season sampling dates occurred between 14 and 20 August. All lakes were ultra-oligotrophic, and represented ranges of depths and sizes characteristic of this area of Greenland [37]. Lake SS32 was not stratified during the study period, and lake SS8 was stratified only during the June sampling trip. All other lakes were stratified during both sampling periods in 2014. Six of the 15 study lakes were sampled previously in 2013, along with six others not included in the 2014 analysis. This subset of lakes were only sampled July 2013 due to logistical constraints, but water collection for methane was consistent with the methods described below. Of the six lakes not re-sampled in 2014, two were in Cluster A, one in Cluster C, and three much more distant from the ice sheet than others (>70 km).

**Table 1:** Characteristics of the 15 lakes used in this study.

Lake	Latitude/Longitude	Region	Distance from the ice sheet (km)	Z_max_ (m)	Surface Area (km^2^)	pH	Total Alkalinity (mEq L^-1^)	Specific Conductance (μS cm^-1^)	DIN (μg L^-1^)
SS32	66.9650 N	A	0	22	0.176	8.6	0.4	52	bd
	-49.8000 W								
SS901	67.1315 N	A	6	15	0.106	7.7	1.0	102	bd
	-50.2350 W								
SS903	67.1297 N	A	4.4	29	0.354	8.0	1.6	191	bd
	-50.1713 W								
SS906	67.1201 N	A	6.7	18	0.085	7.3	0.5	71	bd
	-50.2547 W								
SS10	66.9292 N	B	19.2	28	0.289	8.0	0.5	64	bd
	-50.4243 W								
SS15	66.9188 N	B	19.7	28	0.358	8.4	0.6	63	bd
	-50.4300 W								
SS16	66.9144 N	B	20.3	13	0.033	6.7	0.6	74	bd
	-50.4410 W								
SS16-B	66.9129 N	B	20.7	9	0.022	6.7	0.6	80	53
	-50.4482 W								
SS18	67.1658 N	B	18.3	11	0.091	7.0	1.2	171	bd
	-50.3488 W								
SS1341	66.9905 N	C	44.4	14	0.070	8.7	2.6	373	bd
	-51.1417 W								
SS1381	67.0160 N	C	42.8	19	0.215	7.2	3.5	639	bd
	-51.1184 W								
SS1590	67.0106 N	C	34.7	18	0.243	8.0	1.8	311	6
	-50.9825 W								
SS2	66.9959 N	C	36.9	12	0.368	8.0	2.6	399	bd
	-50.9637 W								
SS8	67.0131 N	C	41.1	10	0.146	7.5	2.5	430	bd
	-51.0758 W								
SS85	66.9823 N	C	47.8	11	0.246	8.6	3.9	652	6
	-51.0559 W								

Values of pH, alkalinity, specific conductance, and dissolved inorganic nitrogen (DIN) are averages over the study period (June and August 2014). Different lake clusters are represented by A, B, and C. Quantification limits were 3 μg L^-1^ for DIN.

### Lake sampling

All lakes were sampled by raft for basic physicochemical data and profiled with a Hydrolab DataSonde 5a (OTT Hydromet, Loveland, CO) to measure water temperature, dissolved oxygen, pH, specific conductance, and location of the thermocline. In stratified lakes, water was taken from the epilimnion, metalimnion, and hypolimnion. In those lakes that were not stratified, we sampled shallow, middle, and bottom waters. For sampling all lakes, we anchored at Z_max_ or at a depth > 1% PAR, based on previous surveys.

Whole water grabs were taken from each lake and each depth using a horizontal Van Dorn bottle. Water samples for ions, DOC, and nutrients were filtered through Whatman GF/F 0.7μm filters and chilled for return and analysis at the University of Maine. Water for chlorophyll *a* (Chl *a*) analysis was also filtered onto GF/F filters and frozen for later analysis.

Methane was sampled from the same depths and at the same time as water taken for samples described above. Acidification and preservation of methane samples has been used in numerous Arctic studies in the past [11, 15, 26] and has consistently demonstrated reliable data. As such, in the field, 10mL of water from each depth was injected into pre-evacuated, He-filled, 20mL scintillation vials acidified with 0.1 mL of 0.1N HCl [15], inverted and returned to the United States for analysis. Due to logistical constraints, only surface water samples were obtained for SS32 and only in June, and only surface water methane samples could be obtained from SS10 on both sampling dates.

### Laboratory methods

Water samples were analyzed for a variety of chemical constituents. Anions (Ca^2+^, Mg^2+^, and Na^+^) were determined by inductively coupled plasma mass spectrometry (ICP-MS) using a Thermo Element 2 (Thermo Fisher Scientific, Inc., Waltham, MA). Sulfate (SO_4_^2-^) concentrations were determined on a Dionex DX500 ion chromatograph (Thermo Fisher Scientific, Inc., Waltham, MA). Ammonium was determined by the phenate method and nitrate by cadmium reduction, both followed by flow injection analysis [45] on a Lachat QuikChem 8500 (Hach Company, Loveland, CO). Quantification limits for both were 3 μg/L. Water for DOC was filtered through pre-combusted 0.7μm GF/F (500°C, 6 hours), and analyzed with an Aurora 1030D TOC analyzer using wet chemical oxidation (OI Analytical, College Station, TX).

Total alkalinity was determined on whole water samples using titration with 0.2N H_2_SO_4_ to pH 4.5 [45]. We determined Chl *a* content on the filters using extraction into 90% acetone followed by centrifugation and analysis on a Varian Cary-50 Ultraviolet-Visible spectrophotometer (Agilent Technologies, Santa Clara, CA) [45]. All Chl *a* samples were measured within three weeks of collection.

Headspace concentrations of methane were determined from scintillation vials of lake water collected in the field using a Shimadzu GC8A (Shimadzu Corp., Tokyo) with flame ionization detector with a 1/8 inch x 1-m molecular sieve 5A column and ultrahigh purity N_2_ carrier gas. Precision of analysis was 10 uL/L = 0.9%, with a detection limit of 0.2 μL/L. Values of methane were corrected for water volume and converted to μmol L^-1^.

### Statistical analysis

Three-way ANOVA was run to determine differences in temperature across regions, sampling periods, and within stratified lake layers. Paired t-tests were run on all data to determine if differences existed between June and August sampling periods. Although lake temperatures varied in this study, no seasonal differences (p > 0.1) were found in our key response variables, therefore data were pooled for further analysis. Given that our lakes were distributed along a transect from the ice sheet, we examined the relationship of lake distance on methane and other physicochemical variables using Spearman correlations (ρ). Additionally, for physicochemical lake variables, Chl *a*, and methane, two-way ANOVA with Tukey HSD post-hoc tests was used to assess differences across the three study clusters (A, B, C), within stratified layers of each lake, along with the interaction of these factors. In cases where data did not meet normality or equal variance assumptions, they were natural log (ln) transformed for analysis.

We used backwards stepwise regression to generate a predictive model for methane concentrations across our lake basins in Greenland. Inputs to the model included physicochemical lake factors (water temperature, dissolved oxygen, Chl *a*, and DOC) and ions (SO_4_^2-^, Na^+^, Mg^2+^, Ca^2+^) known to be correlated with methane in this region. All data were ln-transformed to meet conditions of normality and autocorrelation among independent variables was assessed prior to model generation.

We tested the model predictions using data collected across southwest Greenland during the summer of 2013. Of the 12 lakes from 2013, half of them (n = 6) were not re-sampled in 2014 for the development of the model due to logistical constraints. All statistics were conducted using the SigmaStat data analysis toolpack in SigmaPlot v.12. 5 (Systat Software, San Jose, CA).

## Results

### Lake physico-chemical variables

Our study lakes were ultra-oligotrophic and chemically dilute, and pH ranging from neutral to slightly basic (Table 1). Dissolved inorganic nitrogen (DIN) was generally below detection, except for a few lakes where it mostly exists as NH_4_-N (Table 1). Conductivities varied across the region, but Cluster C lakes generally had the highest values in our sampling region. Alkalinities also tended to be higher in Cluster C, but there was no clear pattern along the sampling transect (Table 1).

**Fig 2:**
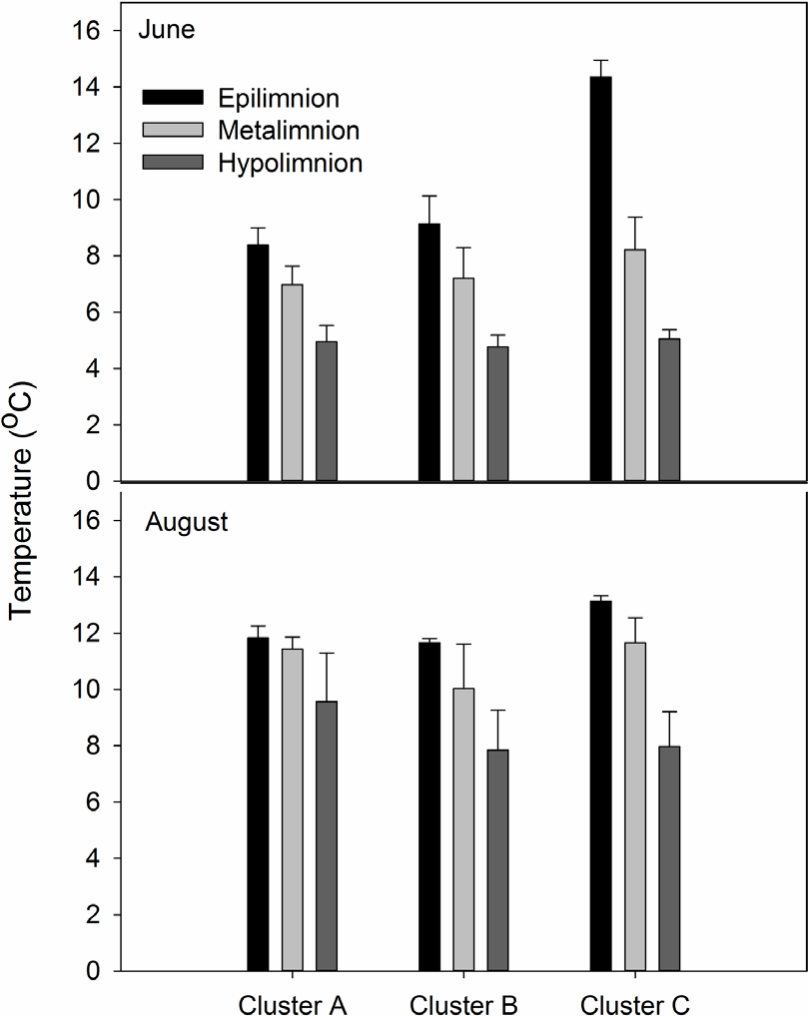
Temperature differences across the ice-free season of summer 2014 in southwest Greenland lakes, including differences in depth and regions. Bars represent 1 SE of the mean. Statistics outlining differences among groups may be found in the text.

Lake temperatures varied across seasons and regions, and within lakes (Fig 2). Cluster C had the warmest lake temperatures overall, while Clusters A and B were cooler but not significantly different from one another (F_2,80_ = 4.1, p = 0.021). In June, the epilimnion, metalimnion, and hypolimnion had significantly different temperatures from one another while in August the hypolimnion only was significantly cooler than the other parts of the lake (Interaction F_2,80_ = 3.9, p = 0.03), as the epilimnion had already substantially eroded by this time.

Ionic composition of lakes varied with distance from the ice sheet. Chloride (ρ = 0.68, p < 0.0001), along with all cations, including Na^+^ (ρ = 0.72, p < 0.0001), Mg ^2+^ (ρ = 0.71, p < 0.0001), and Ca^2+^ (ρ = 0.66, p < 0.0001), were higher in lakes farther from the edge of the ice sheet.

**Fig 3:**
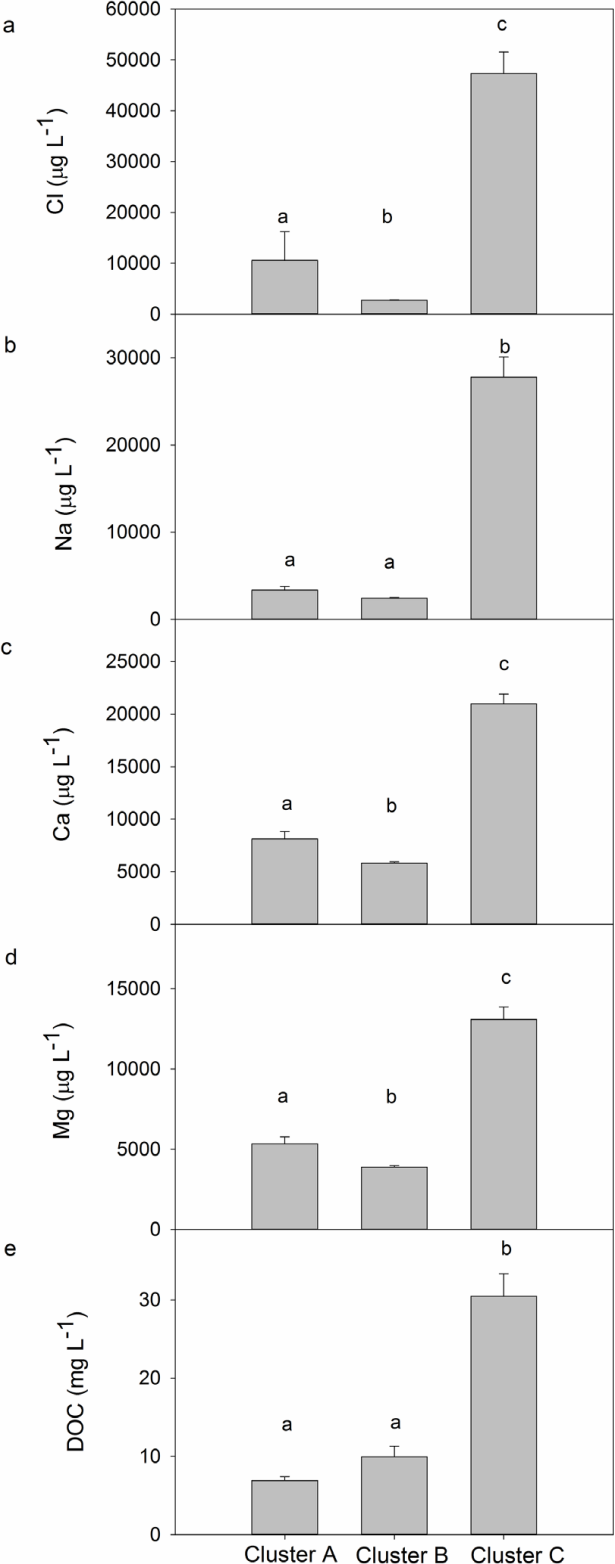
Regional differences in (a) chloride (Cl), (b) sodium (Na), (c) calcium (Ca), (d) magnesium (Mg), and (e) DOC in lakes across southwest Greenland. Bars represent 1 SE of the mean, and letters represent significant differences (p < 0.05) based on post-hoc comparisons of ln-transformed data.

**Fig 4:**
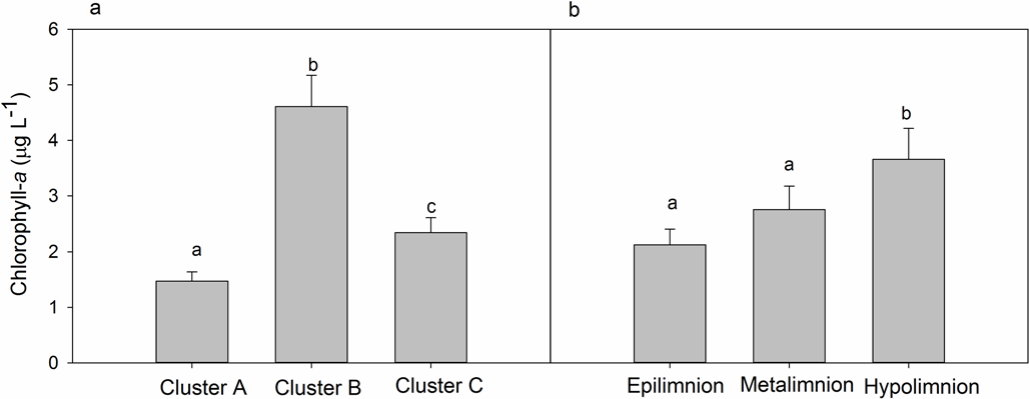
Patterns in chlorophyll-a (a) regionally and (b) within lakes across southwest Greenland. Bars represent 1 SE of the mean, and letters represent significant differences (p < 0.05) based on post-hoc comparisons of ln-transformed data.

Regionally, Cluster C lakes had the highest concentrations of ions and DOC compared to any other regions sampled (Fig 3). Chl *a* in lakes varied among regions, with Cluster B having the highest followed by Cluster C, and Cluster A lakes having the lowest (Fig 4A, F_2,78_ = 21.45, p < 0.001). Chl *a* also increased significantly with depth in lakes (Fig 4B, F_2,78_ = 5.17, p = 0.008), with no interaction between depth and region (F_4,78_ = 0.40, p = 0.83).

**Fig 5:**
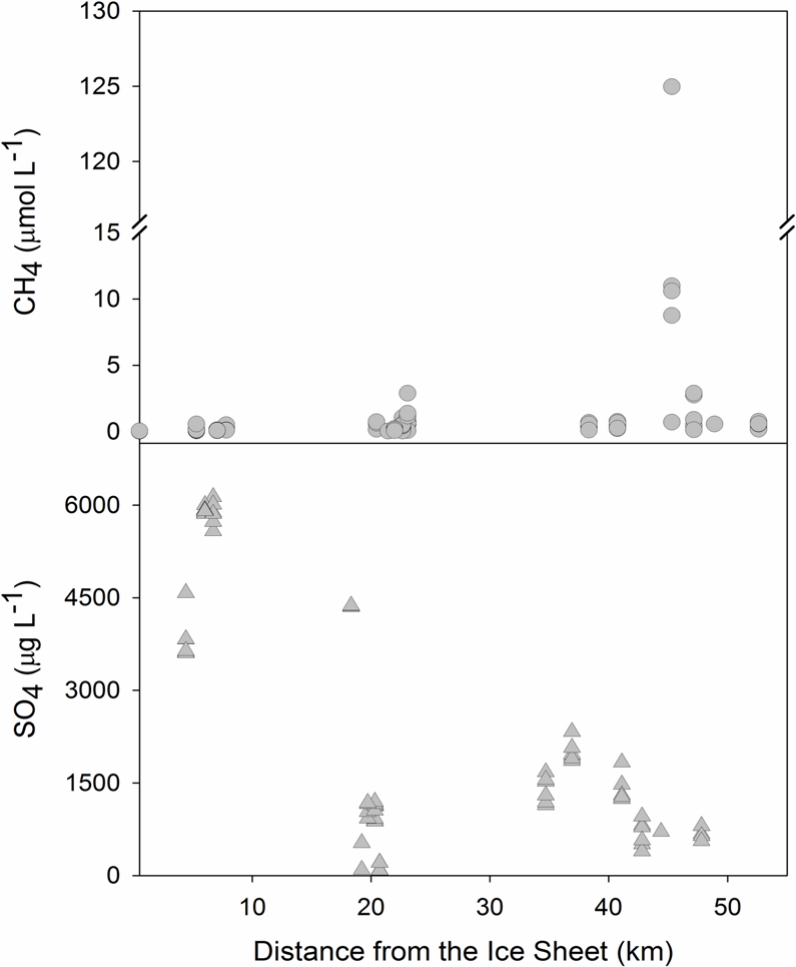
Patterns in lake-water methane and sulfate concentrations across the sampling region from the Greenland Ice Sheet outward in 2014.

Regional differences in lakewater sulfate were also found (F_2,78_ = 32.00, p < 0.001), with concentrations generally decreasing with distance from the ice sheet (ρ = -0.62, p < 0.0001; Fig 5B). Sulfate concentrations (ug L^-1^) were significantly higher in Cluster A (mean = 4707, SE = 265), followed by Cluster C (mean = 1181, SE = 96), and Cluster B lakes (mean = 722, SE = 90; p < 0.001 in all pairwise comparisons between lake clusters.

### Methane

Average in-lake concentrations of methane were 2.53 μmol L^-1^ during the open-water season of 2014, with higher concentrations during June after ice-out compared to mid-August (Table 2) although these differences were not significant (t = 0.70, df = 77, p = 0.49). Methane variability in lakes and across depths was much greater in the earlier (CV = 5.4) compared to late season (CV = 2.0).

**Table 2:** Methane concentrations (μmol L^-1^) across the study region in southwest Greenland.

Lake Region	Sampling Time	Average	SE	Range
Entire Study Area	Whole Season	2.52	1.59	0.02–1.59
	June	3.51	2.84	0.02–124.97
	August	1.27	0.44	0.06–10.60
Cluster A	Whole Season*	0.15	0.03	0.04–0.56
	June	0.16	0.04	0.04–0.56
	August	0.15	0.05	0.06–0.15
Cluster B	Whole Season*	0.56	0.12	0.02–2.88
	June	0.42	0.08	0.02–1.05
	August	0.80	0.27	0.06–2.88
Cluster C	Whole Season*	5.52	4.02	0.10–124.97
	June	8.62	8.04	0.17–124.97
	August	2.22	0.89	0.10–10.60

Significant differences across lake regions (p<0.05) are denoted by the asterisks (*).

In general, lakes closest to the ice sheet had the lowest methane, with concentrations increasing with distance from the ice sheet (ρ = 0.64, p < 0.0001), showing a clear clustering of methane values along the sampling region (Fig 5A). Lakes in Cluster C had significantly higher in-lake methane concentrations, while lakes in Clusters A and B had lower, but quite similar values (Table 2, Fig 5A; F_2,76_ = 17.6, p < 0.0001; p < 0.02 for all pairwise comparisons). Methane concentrations were not different from June to August within each lake region (all p > 0.2). The highest values of methane were found in Cluster C lake SS8, where they reached 125 μmol L^-1^ in June. Given the extremely high methane concentration in one lake (124.97 μmol L^-1^) we ran an additional ANOVA without the high value to compare methane concentration in each cluster, which led to the same results (F_2,75_ = 18.6, p < 0.001; p < 0.001 for all pairwise comparisons) as the previous analysis. Therefore, the full set of methane data were used in further analysis.

### Modeling methane

**Fig 6:**
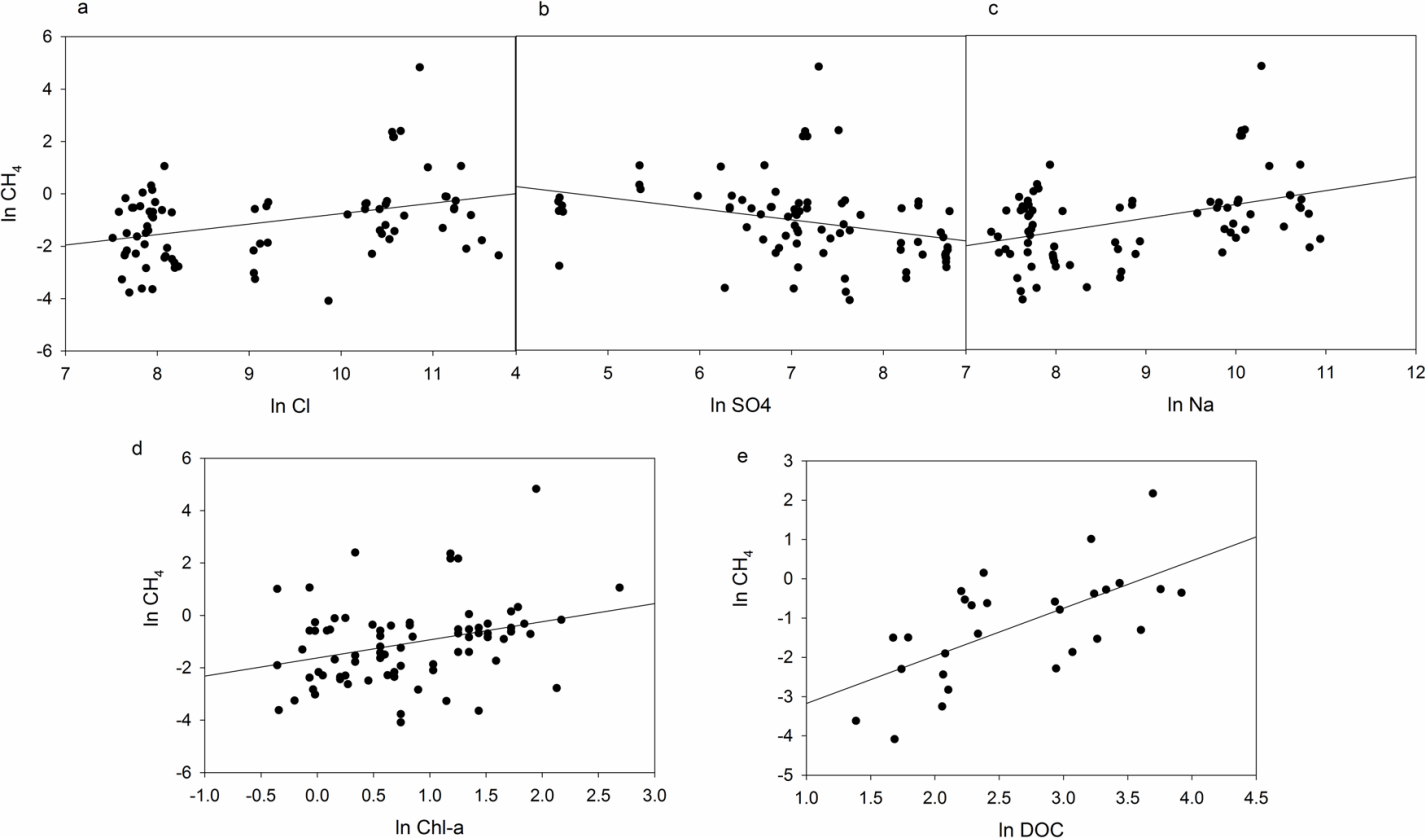
Individual relationships between predictors of methane (CH_4_) in Greenland lakes. All lines represent significant regression models. Statistics for each relationship can be found in Table 3.

For these Greenland lakes, lake-water methane was best predicted by a linear combination of factors including SO_4_, Na, Cl, and Chl *a* (Table 3). Individual linear regressions between CH_4_ and the predictor variables indicated significant relationships, but poor predictive ability individually (Table 3, Fig 6). Only in combination did these predictors explain approximately 75% of the variation in methane concentrations. By itself, DOC explained some variation (Table 3) but only in epilimnetic samples, likely why it was not selected for the full model. The full model above was more robust across lakes, depths, and time.

**Table 3:** Regression results for predicting methane (CH_4_) in southwest Greenland lakes, along with the validation of this model using 2013 pilot lake data.

n	Model	R^2^	F-stat	overall p	individual p	
79	ln CH_4_ = -0.881 (ln Cl) -0.414 (ln SO_4_) + 1.826 (ln Na) + 1.002 (ln Chl-*a*)– 6.756	0.746	F_4,18_ = 13.206	< 0.001	ln Cl	0.03	
					ln SO_4_	0.02	
					ln Na	< 0.001	
					ln Chl-*a*	0.007	
79	ln CH_4_ = - 0.423 (ln SO_4_) + 1.946	0.1	F_1,78_ = 8.493	0.005			
77	ln CH_4_ = 0.692 (ln Chl-*a*) - 1.625	0.1	F_1,76_ = 8.819	0.005			
79	ln CH_4_ = 0.400 (ln Cl) -4.574	0.133	F_1,78_ = 11.803	< 0.001			
79	ln CH_4_ = 0.526 (ln Na) -5.715	0.17	F_1,78_ = 15.729	< 0.001			
28	ln CH_4_ = 1.211 (ln DOC) - 4.387	0.418	F_1,27_ = 18.656	< 0.001			
12	Observed CH_4_ = 0.587(Predicted CH_4_) + 0.345	0.784	F_1,11_ = 36.263	< 0.001			

**Fig 7:**
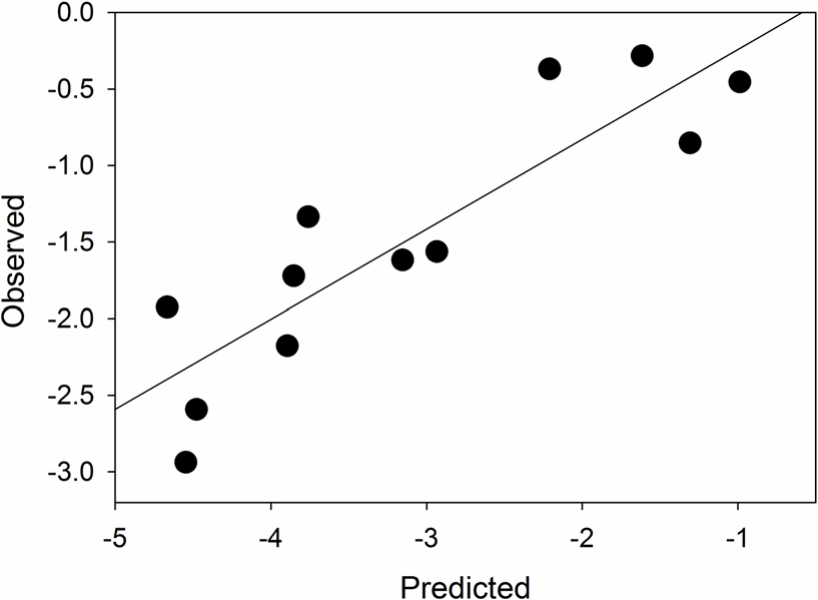
Relationship between observed and predicted lake methane values in pilot lakes sampled in 2013.

There was a strong relationship between measured and predicted values of lake methane (Table 3, Fig 7) from our 2013 sampling, although the model generally underestimated values (Table 4). The best predictions for methane were in lakes SS2 and SS1590, both of which were re-sampled in 2014. In some lakes where the model underperformed (e.g. SS66, SS68), sulfate concentrations were extremely high, much higher than the range of sulfate used to generate our model (0.09–6.14 mg L^-1^). These lakes were also found halfway to the coast, in a region that we were unable to sample in 2014.

**Table 4:** Lake characteristics for pilot lakes sampled in 2013.

	Date	Depth sampled (m)	SO_4_ mg L^-1^	Na mg L^-1^	Chl-*a* μg L^-1^	Cl mg L^-1^	measured CH_4_ (μmol L^-1^)	2014 model CH_4_ (μmol L^-1^)
Lake								
SS 1	19-Jul-13	2	5.0	6.5	3.5	17.1	0.8	0.20
SS 15	30-Jul-13	3	1.1	1.8	1.3	4.9	0.2	0.04
SS 1590	20-Jul-13	2	1.2	14.7	1.9	46.4	0.6	0.37
SS 16	1-Aug-13	2	1.1	2.3	2.9	6.4	0.7	0.11
SS 2	20-Jul-13	3	2.0	21.5	1.2	67.6	0.4	0.27
SS 21	31-Jul-13	3	1.6	1.6	0.8	3.7	0.2	0.02
SS 56	28-Jul-13	3	6.4	12.1	0.7	39.1	0.2	0.05
SS 66	28-Jul-13	3	16.3	3.4	0.6	10.7	0.1	0.01
SS 68	31-Jul-13	3	60.4	15.1	0.3	49.9	0.1	0.01
SS 901	26-Jul-13	3	5.4	2.4	1.1	7.3	0.1	0.02
SS 904	25-Jul-13	3	2.8	2.0	1.1	6.1	0.3	0.02
SS 906	26-Jul-13	3	5.4	1.5	0.8	4.8	0.1	0.01

Data included here were those found to be significant predictors of lake methane concentrations, including sulfate (SO_4_), sodium (Na), chlorophyll-*a* (Chl-*a*), and chloride (Cl).

## Discussion

We found that concentrations of methane in lake basins across the ice-free landscape of southwest Greenland vary greatly, and in some cases were on the high end of ranges seen in other parts of the Arctic (Table 5). Our investigation of these Greenland lakes suggested that, in the short term, ionic and biotic factors were related methane in surface waters. These factors, including ions such as Cl, Na and SO_4_ along with Chl *a* relate to the position of lakes within the landscape, as methane was generally found in higher concentrations in areas farther from the ice sheet. Our mean value of methane fell within ranges reported for other lakes spanning the Northern Hemisphere (Table 5), with a majority of values < 2.0 μmol L^-1^. Most lakes tended to have low water column methane, while higher values have been reported in sediments elsewhere [15] or in anoxic hypolimnia [18, 46]. Although [31] also reported high water column methane in Lake N2 in the Alaskan arctic, this was the result of N and P fertilization experiments, so the results are likely influenced by factors not addressed in this study. Our data are also consistent with values measured in small lakes adjacent to our study areas in southwest Greenland [47, 48]. The highest values of methane in southwest Greenland occur under the ice [48] or during spring turnover [49].

**Table 5:** Ranges of methane in freshwater lakes across the Northern Hemisphere.

CH_4_ range (umol L^-1^)	Location	Reference
0.58–3.16	Lake Washington, USA	[14]
0.8–1.5 (water column)	North Slope, Alaska (AK)	[15]
5.8–149 (surficial sediment)	North Slope, Alaska (AK)	[15]
0.03–160	south central Sweden	[18]
0.02–0.82	North Slope, AK	[23]
0.10–0.50	Lac Cromwell, Canada	[24]
0.13–165	North Slope, AK	[31]
1.0–20.6	Finland	[46]
0.11–0.12	southwest Greenland	[47]
0.9–220	southwest Greenland	[48]
0.02–120	Northern Canada	[64]
0.1–1.6	Rocky Mountains, USA	[68]
<0.10–63.9	Wisconsin, USA	[69]
0.27–2.32	Wisconsin, USA	[70]
0.08–1.9	south central Sweden	[70]
0.2–1.0	Canadian Arctic	[71]
0.02–125	southwest Greenland	This Study

Landscape position was a factor influencing methane across Greenland as seen by significant regional differences in not only methane, but also the physico-chemical and biological factors that ultimately were related to its availability. The Greenland Ice Sheet is a major feature of this landscape, and has both direct and indirect influences on the biogeochemistry and ecology of lakes in this region. Lake ontogeny in Greenland is directly related to the timing of ice sheet regression from a given region [39], resulting in older lakes with higher dissolved ions in lakes farther from the ice sheet [37, 50, 51]. The lack of hydrologic connectivity between many of the lakes in this region creates endorheic basins with geochemical signals that can potentially act as proxies for other biogeochemical processes. Another study [48] in the same region of Greenland, offers additional support that landscape position plays a key role in regulating methane in Greenland lakes due to variation in geochemistry driving changes to methanogenesis and methane oxidation in their study lakes. Further, research from the North Slope of Alaska has demonstrated high levels of methanogenesis, methane efflux, and pore water methane [11, 15, 25, 26] in lakes GTH 112 and 114, which lie on the oldest exposed till in the region [52]. Conversely, patterns of increasing lake sulfate [53] are potentially related to increases in biogenic sulfate deposition over the last decade [54], which are unrelated to ice sheet processes. Given the relationships found in this study, it is reasonable to conclude that the importance of ions in our predictive models serve as a proxy for age and watershed composition, not as a factor that directly influences the concentration of methane in our study lakes.

Biogeochemically-speaking, lakes farther from the ice sheet are likely more ideal for methane production for a variety of factors. First, the greatest concentrations of methane were seen in the lakes of Cluster C, which were not only farther from the ice sheet, but also were the warmest lakes in our survey. While our methane data were significantly correlated with temperature (ρ = 0.22, p = 0.04), the relationship was fairly weak, likely a consequence of our short-term data collection. Numerous studies have noted the importance of temperature on methane dynamics (e.g. [55, 56, 57]). Moreover, multiple studies have predicted higher methane flux in a warming climate [7, 58], a likely future outcome in Greenland as well, given the intensity of recent warming in the region [2].

Second, the Greenland Ice Sheet indirectly influences the terrestrial ecology of the region through the development of soils and more complex plant communities farther from the ice edge (e.g. succession [39, 59]). As such, lakes with more complex terrestrial communities surrounding them will likely receive greater inputs of terrestrial carbon, as demonstrated by significantly higher DOC in lakes of Cluster C. Previous work in Greenland has noted the terrestrial origin of lake DOC [53]. While previous studies have demonstrated a strong relationship between permafrost melt and the import of labile C into aquatic systems (e.g. [9, 60]), there are likely multiple landscape and in-lake processes that can supplement methane production. In our study, the strongest individual predictor of methane was lakewater DOC, consistent with other work in the Arctic (e.g. [61, 62]). While many of the highest fluxes of methane from the Arctic come from organic rich, peat or yedoma-dominated shallow ponds [49], our data suggest that lakes in Greenland process C differently since they are not underlain by such C-rich precursors. Further, lakes in southwest Greenland have demonstrated significant losses in DOC over the last decade [53], have very low C burial efficiencies of around 22% [63], and have sediments with relatively low organic matter (< 30%, N.J. Anderson, personal communication). In spite of a strong relationship between DOC and CH_4_ in the epilimnia of our lakes, it was not selected as an important predictor in the larger model. A more comprehensive inventory of DOC within the lakes and across the growing season may demonstrate a stronger relationship.

Third, aquatic primary production may supplement carbon resources needed by methanogens, as seen in some lakes of Cluster B, where Chlorophyll-a concentrations were significantly higher than elsewhere, but yet methane concentrations were on a similar scale to most lakes in Cluster C that were farther away. Recent studies have suggested a strong association between methanogenesis and phytoplankton in Arctic lakes [24, 48], as the pelagic algae are likely providing a C-source to fuel methane production in the water column. In a recent study at Toolik Lake, AK [23], terrestrial methanogensis and export into lake waters were found to be significant contributors to lake concentrations of methane. Groundwater wells installed near some of our lakes indicated large flushes of nutrients and particulates after ice-off and sporadically over the growing season (Northington and Saros, unpublished data). Future studies should investigate the extent to which terrestrial inputs of methane or methane precursors supplement lake methane production as compared to autochthonous production.

A recent analysis of methane in the Arctic [49] has noted that the greatest annual fluxes of methane are released at ice-off (~23%) and during mixing periods. This has been verified by work in Greenland which found concentrations of methane under ice ranging from 50–220 μmol L^-1^ [48]. Typically, methane has been found in the anoxic bottom waters of lakes near sediments [15, 18, 64] as methanogensis is a primarily anaerobic process [65, 66], but this pattern was not always seen in our lakes. For example, in Cluster C, the hypolimnion of Lake SS8 had the highest value of methane in this study (125 μmol L^-1^), while Lake SS1590 had one of the lowest (0.1 μmol L^-1^) in spite of both being strongly stratified with anoxic bottom waters. Lake SS8 was also notable for having consistently high methane throughout lake depths in both June (0.7–125 μmol L^-1^), during stratified conditions, and in August (8.8–10.6 μmol L^-1^) after turnover. Lake SS1590 actually demonstrated higher methane in the oxygenated surface waters (0.7 μmol L^-1^). Most of our data collection occurred after stratification had set up in most of our lakes, therefore our methane measures likely occurred after the greatest losses of seasonal methane in these small kettle lakes [49, 67].

## Conclusions

In spite of the short duration of our study, our model provides some intriguing possibilities for future studies of methane in Greenland, and the Arctic in general. While our model underestimated data from our 2013 pilot study, it reinforces the need to further examine multiple factors across the landscape that work synergistically to influence methane. In the 2013 data for example, Lakes 66 and 68 were in a region halfway between Kangerlussuaq and the coast noted for having modest (~11%) increases in lake sulfate over the past decade [48]. Even so, the concentrations of sulfate in this region are extremely high compared to other lakes sampled outside of the range of those used in the 2014 model.

Our data represent the most extensive reporting the methane across Greenland lakes of which we are aware. Even so, we caution that these data represent a relatively low-resolution view of the dynamic nature of methane in Greenland, and the Arctic in general. As with many other short-term studies of methane dynamics in the Arctic, our results likely underestimate seasonal contributions of lake methane in the region [67]. More comprehensive, long term data in the region would likely demonstrate significant fluxes of methane after ice out [49], especially given recent research in Greenland indicating concentrations > 200 μM under the ice [48]. High frequency methane and temperature data would elucidate the patterns of temperature-driven methanogensis [55, 56, 57] during the summer open-water period. Given the lack of hydrologic connectivity between most lakes in this part of Greenland, geochemical signatures of surface waters, in addition to distance from the ice sheet, may be important factors to consider in future studies of methane in this region. We regard this work as a first step in understanding methane dynamics in a rapidly-changing, yet relatively understudied part of the Arctic.

## Supporting Information

S1 Table Physical and biological data collected from southwestern Greenland lakes during summer 2014. Lake cluster refers to the sampling regions outlined in the Materials and Methods. Ice sheet distance (Dist.) is measured as the linear distance from the edge of the Greenland Ice Sheet westward toward the lake.
(DOCX)

S2 Table Chemical data from Greenland lakes sampled during summer 2014. Specific details related to analytical techniques may be found in the Materials and Methods.
(DOCX)
