# Supplementary material for: Factors Controlling Methane in Arctic Lakes of Southwest Greenland
Source: PLoS One. 2016 Jul 25;11(7):e0159642. doi: 10.1371/journal.pone.0159642 (PMC4959701; doi:10.1371/journal.pone.0159642)
Supplement: S1 Table — Lake cluster refers to the sampling regions outlined in the Materials and Methods. Ice sheet distance (Dist.) is measured as the linear distance from the edge of the Greenland Ice Sheet westward toward the lake. (DOCX) [file pone.0159642.s001.docx]

**S1 Table. Physical and biological data collected from southwestern Greenland lakes during summer 2014.** Lake cluster refers to the sampling regions outlined in the Materials and Methods. Ice sheet distance (Dist.) is measured as the linear distance from the edge of the Greenland Ice Sheet westward toward the lake.

| Lake | Date | Lake Cluster | Lake Depth (m) | Ice Sheet Dist (km) | Temp (°C) | Chl-*a* (µg L^-1^) | Dissolved Oxygen (mg L^-1^) |
| --- | --- | --- | --- | --- | --- | --- | --- |
| Control | 6/14/2014 | B | 2 | 20.7 | 8.6 | 4.6 | 11.3 |
| Control | 6/14/2014 | B | 4 | 20.7 | 6.0 | 8.8 | 11.3 |
| Control | 6/14/2014 | B | 7 | 20.7 | 3.5 | 6.7 | 2.2 |
| ss16 | 6/15/2014 | B | 2 | 20.3 | 5.4 | 3.9 | 11.1 |
| ss16 | 6/15/2014 | B | 5 | 20.3 | 5.4 | 3.5 | 11.4 |
| ss16 | 6/15/2014 | B | 8 | 20.3 | 4.7 | 3.9 | 11.1 |
| ss18 | 6/18/2014 | C | 1 | 18.3 | 16.6 | 2.8 | 10.5 |
| ss18 | 6/18/2014 | C | 3 | 18.3 | 12.8 | 4.2 | 10.6 |
| ss18 | 6/18/2014 | C | 6 | 18.3 | 5.9 | 6.3 | 10.7 |
| ss1381 | 6/20/2014 | C | 2 | 42.8 | 13.7 | 0.7 | 11.0 |
| ss1381 | 6/20/2014 | C | 5 | 42.8 | 7.6 | 0.9 | 10.8 |
| ss1381 | 6/20/2014 | C | 9 | 42.8 | 4.5 | 2.3 | 8.9 |
| ss8 | 6/20/2014 | C | 1 | 41.1 | 15.6 | 1.6 | 11.5 |
| ss8 | 6/20/2014 | C | 4 | 41.1 | 7.1 | 1.4 | 6.0 |
| ss8 | 6/20/2014 | C | 7 | 41.1 | 4.0 | 7.0 | 0.8 |
| ss906 | 6/22/2014 | A | 2 | 6.7 | 10.4 | 0.7 | 11.4 |
| ss906 | 6/22/2014 | A | 5 | 6.7 | 5.4 | 1.2 | 11.9 |
| ss906 | 6/22/2014 | A | 10 | 6.7 | 4.2 | 3.5 | 10.7 |
| ss901 | 6/22/2014 | A | 2 | 6.0 | 8.6 | 1.2 | 12.0 |
| ss901 | 6/22/2014 | A | 5 | 6.0 | 6.7 | 0.9 | 12.2 |
| ss901 | 6/22/2014 | A | 9 | 6.0 | 4.5 | 1.2 | 11.8 |
| ss1590 | 6/25/2014 | C | 1 | 34.7 | 14.2 | 1.8 | 10.4 |
| ss1590 | 6/25/2014 | C | 4 | 34.7 | 6.9 | 3.9 | 11.0 |
| ss1590 | 6/25/2014 | C | 8 | 34.7 | 4.7 | 4.9 | 8.8 |
| ss2 | 6/25/2014 | C | 2 | 36.9 | 13.3 | 1.4 | 10.8 |
| ss2 | 6/25/2014 | C | 6 | 36.9 | 6.7 | 1.8 | 11.5 |
| ss2 | 6/25/2014 | C | 9 | 36.9 | 5.2 | 3.9 | 11.2 |
| Control | 6/26/2014 | B | 2 | 20.7 | 12.8 | 4.2 | 10.4 |
| Control | 6/26/2014 | B | 4 | 20.7 | 7.1 | 5.6 | 10.4 |
| Control | 6/26/2014 | B | 7 | 20.7 | 3.9 | 8.4 | 1.9 |
| ss16 | 6/26/2014 | B | 2 | 20.3 | 10.5 | 3.5 | 10.5 |
| ss16 | 6/26/2014 | B | 5 | 20.3 | 10.3 | 3.9 | 10.6 |
| ss16 | 6/26/2014 | B | 8 | 20.3 | 5.3 | 2.1 | 9.6 |
| ss16 | 6/26/2014 | B | 10 | 20.3 | 4.8 | 4.2 | 5.5 |
| ss15 | 6/26/2014 | B | 3 | 19.7 | 8.9 | 1.9 | 11.5 |
| ss15 | 6/26/2014 | B | 9 | 19.7 | 6.4 | 1.8 | 11.7 |
| ss32 | 6/26/2014 | A | 2 | 0.0 | 7.2 | 2.1 | 11.9 |
| ss32 | 6/26/2014 | A | 6 | 0.0 | 7.2 | 2.1 | 12.0 |
| ss32 | 6/26/2014 | A | 14 | 0.0 | 6.7 | 3.2 | 11.9 |
| ss10 | 6/26/2014 | B | surface | 19.2 | 8.6 | 0.7 |  |
| ss86 | 6/26/2014 | A | surface | 19.2 | 7.0 | 1.1 |  |
| ss903 | 6/27/2014 | A | 3 | 4.4 | 8.7 | 0.8 | 11.9 |
| ss903 | 6/27/2014 | A | 8 | 4.4 | 8.6 | 1.0 | 12.0 |
| ss903 | 6/27/2014 | A | 16 | 4.4 | 4.4 | 1.2 | 8.9 |
| ss85 | 6/28/2014 | C | 2 | 47.8 | 12.7 | 0.9 |  |
| ss85 | 6/28/2014 | C | 6 | 47.8 | 6.0 | 1.4 |  |
| ss1590 | 8/14/2014 | C | 3 | 34.7 | 12.7 | 2.3 | 10.3 |
| ss1590 | 8/14/2014 | C | 8 | 34.7 | 9.8 | 1.8 | 8.6 |
| ss1590 | 8/14/2014 | C | 11 | 34.7 | 7.0 | 1.1 | 6.6 |
| ss2 | 8/14/2014 | C | 3 | 36.9 | 13.4 | 2.3 | 10.5 |
| ss2 | 8/14/2014 | C | 8 | 36.9 | 12.8 | 1.9 | 10.1 |
| ss2 | 8/14/2014 | C | 10 | 36.9 | 6.7 | 1.8 | 6.7 |
| ss16 | 8/15/2014 | B | 2 | 20.3 | 11.9 | 5.6 | 10.1 |
| ss16 | 8/15/2014 | B | 5 | 20.3 | 11.9 | 5.3 | 10.1 |
| ss16 | 8/15/2014 | B | 8 | 20.3 | 11.8 | 4.6 | 10.1 |
| ss16 | 8/15/2014 | B | 10 | 20.3 | 7.5 | 4.6 | 1.5 |
| control | 8/15/2014 | B | 2 | 20.7 | 11.7 | 5.6 | 10.5 |
| control | 8/15/2014 | B | 4 | 20.7 | 11.3 | 6.0 | 10.6 |
| control | 8/15/2014 | B | 7 | 20.7 | 6.9 | 14.7 | 2.1 |
| ss15 | 8/16/2014 | B | 3 | 19.7 | 11.4 | 1.8 | 11.9 |
| ss15 | 8/16/2014 | B | 11 | 19.7 | 6.9 | 2.1 | 12.0 |
| ss15 | 8/16/2014 | B | 14 | 19.7 | 5.2 | 2.5 | 11.9 |
| ss1341 | 8/17/2014 | C | 3 | 44.4 | 12.5 | 0.9 | 10.6 |
| ss1381 | 8/17/2014 | C | 2 | 42.8 | 13.3 | 1.2 | 10.6 |
| ss1381 | 8/17/2014 | C | 8 | 42.8 | 9.2 | 1.3 | 10.2 |
| ss1381 | 8/17/2014 | C | 11 | 42.8 | 5.5 | 2.8 | 7.5 |
| ss8 | 8/17/2014 | C | 2 | 41.1 | 13.1 | 3.3 | 10.3 |
| ss8 | 8/17/2014 | C | 5 | 41.1 | 13.0 | 3.5 | 10.1 |
| ss8 | 8/17/2014 | C | 7 | 41.1 | 12.6 | 3.3 | 9.8 |
| ss85 | 8/18/2014 | C | 2 | 47.8 | 13.8 | 1.0 | 10.7 |
| ss85 | 8/18/2014 | C | 6 | 47.8 | 13.5 | 1.0 | 10.7 |
| ss85 | 8/18/2014 | C | 9 | 47.8 | 8.1 | 1.1 | 8.1 |
| ss903 | 8/19/2014 | A | 3 | 4.4 | 11.0 | 0.7 | 11.4 |
| ss903 | 8/19/2014 | A | 10 | 4.4 | 10.6 | 1.0 | 11.2 |
| ss903 | 8/19/2014 | A | 18 | 4.4 | 6.1 | 1.1 | 11.7 |
| ss906 | 8/20/2014 | A | 2 | 6.7 | 12.4 | 1.3 |  |
| ss906 | 8/20/2014 | A | 5 | 6.7 | 12.0 | 2.0 |  |
| ss906 | 8/20/2014 | A | 10 | 6.7 | 11.3 | 2.0 |  |
| ss901 | 8/20/2014 | A | 2 | 6.0 | 12.1 | 1.0 |  |
| ss901 | 8/20/2014 | A | 5 | 6.0 | 11.7 | 1.6 |  |
| ss901 | 8/20/2014 | A | 10 | 6.0 | 11.3 | 1.3 |  |
|  |  |  |  |  |  |  |  |
