# Supplementary material for: Factors Controlling Methane in Arctic Lakes of Southwest Greenland
Source: PLoS One. 2016 Jul 25;11(7):e0159642. doi: 10.1371/journal.pone.0159642 (PMC4959701; doi:10.1371/journal.pone.0159642)
Supplement: S2 Table — Specific details related to analytical techniques may be found in the Materials and Methods. (DOCX) [file pone.0159642.s002.docx]

**S2 Table. Chemical data from Greenland lakes sampled during summer 2014.** Specific details related to analytical techniques may be found in the Materials and Methods.

| Lake | Date | Lake Cluster | Lake Depth | Chloride (µg L^-1^) | Sulfate (µg L^-1^) | Sodium (µg L^-1^) | Magnesium (µg L^-1^) | Calcium (µg L^-1^) | Methane (ppm) | Methane (µmol L^-1^) | DOC (mg L^-1^) |
| --- | --- | --- | --- | --- | --- | --- | --- | --- | --- | --- | --- |
| Control | 6/14/2014 | B | 2 | 2910.4 | 86.5 | 2181.4 | 3888.3 | 5998.7 | 11.7 | 0.7 | 9.1 |
| Control | 6/14/2014 | B | 4 | 2104.6 | 87.7 | 1979.2 | 3481.0 | 4988.8 | 13.5 | 0.8 |  |
| Control | 6/14/2014 | B | 7 | 3488.0 | 91.1 | 3208.9 | 5315.6 | 6816.1 | 7.9 | 0.5 |  |
| ss16 | 6/15/2014 | B | 2 | 2296.6 | 884.5 | 2091.5 | 3774.4 | 5587.7 | 9.4 | 0.6 | 9.3 |
| ss16 | 6/15/2014 | B | 5 | 2263.6 | 876.6 | 2055.8 | 3690.7 | 5484.2 | 9.4 | 0.6 |  |
| ss16 | 6/15/2014 | B | 8 | 2538.9 | 923.6 | 2322.2 | 3838.2 | 5706.3 | 16.8 | 1.0 |  |
| ss18 | 6/18/2014 | C | 1 | 9914.5 | 4353.0 | 7585.1 | 7557.4 | 12940.2 | 2.5 | 0.2 | 21.6 |
| ss18 | 6/18/2014 | C | 3 | 9777.4 | 4366.2 | 6944.6 | 7302.2 | 13121.0 | 10.0 | 0.6 |  |
| ss18 | 6/18/2014 | C | 6 | 9971.2 | 4375.2 | 6973.0 | 7319.7 | 13348.4 | 11.7 | 0.7 |  |
| ss1381 | 6/20/2014 | C | 2 | 56724.9 | 508.0 | 31923.7 | 14734.9 | 19997.9 | 44.0 | 2.8 | 24.9 |
| ss1381 | 6/20/2014 | C | 5 | 81400.0 | 816.5 | 45032.8 | 20299.3 | 25302.3 | 46.4 | 2.9 |  |
| ss1381 | 6/20/2014 | C | 9 | 90720.2 | 785.4 | 49541.9 | 22074.9 | 27583.6 | 7.1 | 0.4 |  |
| ss8 | 6/20/2014 | C | 1 | 29105.6 | 1305.3 | 16600.9 | 10636.8 | 21351.7 | 11.2 | 0.7 | 50.4 |
| ss8 | 6/20/2014 | C | 4 | 42210.7 | 1833.9 | 24328.5 | 13511.6 | 29498.8 | 176.4 | 11.0 |  |
| ss8 | 6/20/2014 | C | 7 | 51953.1 | 1475.8 | 29241.1 | 16125.8 | 37669.8 | 2004.5 | 125.3 |  |
| ss906 | 6/22/2014 | A | 2 | 2134.9 | 5582.9 | 1451.0 | 3692.8 | 7658.7 | 3.6 | 0.2 | 5.3 |
| ss906 | 6/22/2014 | A | 5 | 1831.3 | 5735.1 | 1563.1 | 3538.3 | 6247.4 | 3.0 | 0.2 |  |
| ss906 | 6/22/2014 | A | 10 | 1959.9 | 6135.5 | 1717.6 | 3788.7 | 6404.3 | 8.0 | 0.5 |  |
| ss901 | 6/22/2014 | A | 2 | 3222.0 | 5884.5 | 2888.8 | 5255.6 | 6794.0 | 1.4 | 0.1 | 7.9 |
| ss901 | 6/22/2014 | A | 5 | 3256.6 | 5865.2 | 2879.0 | 5645.4 | 7001.5 | 1.5 | 0.1 |  |
| ss901 | 6/22/2014 | A | 9 | 3315.2 | 6004.4 | 2924.2 | 5609.7 | 6968.3 | 2.0 | 0.1 |  |
| ss1590 | 6/25/2014 | C | 1 | 23646.5 | 1142.8 | 14339.3 | 8126.3 | 14245.0 | 7.3 | 0.5 | 19.5 |
| ss1590 | 6/25/2014 | C | 4 | 33648.9 | 1522.6 | 19409.6 | 10594.3 | 19506.4 | 4.0 | 0.2 |  |
| ss1590 | 6/25/2014 | C | 8 | 37315.2 | 1676.2 | 22030.5 | 11697.5 | 20779.9 | 2.8 | 0.2 |  |
| ss2 | 6/25/2014 | C | 2 | 34373.7 | 1860.9 | 20782.0 | 11456.2 | 22218.9 | 3.5 | 0.2 | 26.1 |
| ss2 | 6/25/2014 | C | 6 | 35766.3 | 1937.2 | 21392.2 | 11429.8 | 22598.9 | 4.9 | 0.3 |  |
| ss2 | 6/25/2014 | C | 9 | 43817.0 | 2328.9 | 25909.5 | 13602.6 | 27176.1 | 7.0 | 0.4 |  |
| Control | 6/26/2014 | B | 2 | 2739.2 | 87.0 | 2016.0 | 3985.6 | 6807.8 | 8.1 | 0.5 | 9.8 |
| Control | 6/26/2014 | B | 4 | 2471.7 | 90.0 | 2199.7 | 3819.2 | 5570.1 | 10.0 | 0.6 |  |
| Control | 6/26/2014 | B | 7 | 3746.4 | 87.3 | 3483.7 | 5220.7 | 7240.6 | 1.0 | 0.1 |  |
| ss16 | 6/26/2014 | B | 2 | 2698.0 | 1166.4 | 2264.6 | 4088.2 | 6074.1 | 4.0 | 0.2 | 10.3 |
| ss16 | 6/26/2014 | B | 5 | 2710.7 | 1129.9 | 2298.2 | 4007.2 | 5988.2 |  | 0.0 |  |
| ss16 | 6/26/2014 | B | 8 | 2669.1 | 1137.0 | 2301.2 | 3922.1 | 5875.1 | 4.6 | 0.3 |  |
| ss16 | 6/26/2014 | B | 10 | 2824.2 | 1123.8 | 2404.1 | 4201.9 | 6238.9 | 0.4 | 0.0 |  |
| ss15 | 6/26/2014 | B | 3 | 2357.1 | 924.5 | 2176.9 | 3417.9 | 5321.7 | 1.6 | 0.1 | 19.0 |
| ss15 | 6/26/2014 | B | 9 | 2387.2 | 1031.7 | 2237.0 | 3511.2 | 5423.3 | 3.1 | 0.2 |  |
| ss32 | 6/26/2014 | A | 2 | 19234.8 | 2069.0 | 2057.5 | 2973.0 | 4289.3 | 0.3 | 0.0 | 5.4 |
| ss32 | 6/26/2014 | A | 6 | 2196.0 | 1981.9 | 2017.4 | 3038.7 | 4190.4 | 0.4 | 0.0 |  |
| ss32 | 6/26/2014 | A | 14 | 2026.7 | 1963.0 | 1939.5 | 3015.3 | 4143.4 | 0.6 | 0.0 |  |
| ss10 | 6/26/2014 | B | surface | 2515.1 | 530.0 | 4204.0 | 3108.7 | 4893.3 | 0.4 | 0.0 | 4.0 |
| ss86 | 6/26/2014 | A | surface | 20416.0 | 95.7 | 14833.4 | 15957.9 | 15293.6 |  | 0.0 | 13.4 |
| ss903 | 6/27/2014 | A | 3 | 8630.6 | 3828.6 | 6087.0 | 8518.9 | 12989.6 | 0.6 | 0.0 | 7.8 |
| ss903 | 6/27/2014 | A | 8 | 8520.2 | 3831.1 | 6164.1 | 8193.2 | 12993.8 | 0.8 | 0.0 |  |
| ss903 | 6/27/2014 | A | 16 | 122646.6 | 4580.6 | 7217.6 | 8784.4 | 13881.4 | 1.5 | 0.1 |  |
| ss85 | 6/28/2014 | C | 2 | 66704.2 | 673.2 | 37551.4 | 17204.8 | 20045.3 | 4.4 | 0.3 | 36.7 |
| ss85 | 6/28/2014 | C | 6 | 102152.4 | 805.7 | 56246.0 | 25072.0 | 28784.0 | 2.7 | 0.2 |  |
| ss1590 | 8/14/2014 | C | 3 | 28730.2 | 1188.2 | 18259.1 | 8022.4 | 13405.9 | 10.9 | 0.7 | 25.5 |
| ss1590 | 8/14/2014 | C | 8 | 28633.3 | 1299.5 | 17868.7 | 8817.7 | 15195.0 | 8.9 | 0.6 |  |
| ss1590 | 8/14/2014 | C | 11 | 30859.2 | 1553.7 | 18943.4 | 8885.6 | 17324.7 | 1.6 | 0.1 |  |
| ss2 | 8/14/2014 | C | 3 | 36311.9 | 1966.1 | 22664.8 | 10621.1 | 19897.0 | 12.1 | 0.8 | 28.0 |
| ss2 | 8/14/2014 | C | 8 | 35856.8 | 1899.0 | 22447.9 | 10795.9 | 19160.3 | 10.8 | 0.7 |  |
| ss2 | 8/14/2014 | C | 10 | 39452.7 | 2070.8 | 24479.8 | 11478.0 | 21784.1 | 3.9 | 0.2 |  |
| ss16 | 8/15/2014 | B | 2 | 3134.0 | 1133.2 | 2092.3 | 3770.0 | 7014.7 | 8.6 | 0.5 | 11.1 |
| ss16 | 8/15/2014 | B | 5 | 2844.8 | 1056.7 | 2191.4 | 3829.6 | 6072.5 | 6.5 | 0.4 |  |
| ss16 | 8/15/2014 | B | 8 | 2799.8 | 1160.7 | 2228.0 | 3777.3 | 5928.3 | 7.0 | 0.4 |  |
| ss16 | 8/15/2014 | B | 10 | 2840.6 | 1196.6 | 2295.2 | 3736.3 | 5852.2 | 8.1 | 0.5 |  |
| control | 8/15/2014 | B | 2 | 2819.5 | 211.8 | 2456.3 | 3883.1 | 5445.6 | 18.6 | 1.2 | 10.8 |
| control | 8/15/2014 | B | 4 | 2787.5 | 209.1 | 2416.4 | 3799.7 | 5365.8 | 22.0 | 1.4 |  |
| control | 8/15/2014 | B | 7 | 3217.3 | 209.6 | 2790.4 | 4419.9 | 5979.2 | 46.2 | 2.9 |  |
| ss15 | 8/16/2014 | B | 3 | 2627.0 | 1179.5 | 2194.8 | 3524.6 | 5236.7 | 3.6 | 0.2 | 6.0 |
| ss15 | 8/16/2014 | B | 11 | 2593.3 | 1161.9 | 2183.9 | 3444.8 | 5129.6 | 2.3 | 0.1 |  |
| ss15 | 8/16/2014 | B | 14 | 2635.4 | 1179.2 | 2272.3 | 3511.6 | 5276.4 | 0.9 | 0.1 |  |
| ss1341 | 8/17/2014 | C | 3 | 33525.3 | 709.3 | 20030.9 | 9015.8 | 22453.9 | 8.9 | 0.6 | 18.8 |
| ss1381 | 8/17/2014 | C | 2 | 69518.5 | 395.0 | 40371.3 | 15042.7 | 19613.7 | 14.3 | 0.9 | 31.1 |
| ss1381 | 8/17/2014 | C | 8 | 68722.5 | 572.3 | 40140.3 | 14751.3 | 18955.3 | 14.5 | 0.9 |  |
| ss1381 | 8/17/2014 | C | 11 | 86189.5 | 958.4 | 49968.1 | 18579.4 | 24332.0 | 2.0 | 0.1 |  |
| ss8 | 8/17/2014 | C | 2 | 39155.2 | 1303.1 | 23106.7 | 11317.2 | 20474.6 | 140.4 | 8.8 | 40.3 |
| ss8 | 8/17/2014 | C | 5 | 38895.1 | 1244.1 | 23533.8 | 11111.9 | 20370.5 | 140.5 | 8.8 |  |
| ss8 | 8/17/2014 | C | 7 | 38439.8 | 1273.1 | 23436.3 | 11464.1 | 20473.6 | 170.0 | 10.6 |  |
| ss85 | 8/18/2014 | C | 2 | 76887.9 | 640.8 | 45651.0 | 17222.3 | 21224.7 | 12.3 | 0.8 | 42.8 |
| ss85 | 8/18/2014 | C | 6 | 75413.3 | 559.5 | 45252.9 | 17615.5 | 21269.7 | 8.9 | 0.6 |  |
| ss85 | 8/18/2014 | C | 9 | 75571.4 | 559.5 | 44635.9 | 18230.9 | 20269.1 | 9.3 | 0.6 |  |
| ss903 | 8/19/2014 | A | 3 | 9119.9 | 3616.4 | 5761.1 | 7522.0 | 12596.0 | 2.4 | 0.1 | 8.0 |
| ss903 | 8/19/2014 | A | 10 | 8502.8 | 3604.4 | 5937.6 | 7693.2 | 12536.8 | 1.8 | 0.1 |  |
| ss903 | 8/19/2014 | A | 18 | 8616.2 | 3641.8 | 6063.1 | 7836.5 | 12603.4 | 9.0 | 0.6 |  |
| ss906 | 8/20/2014 | A | 2 | 2095.8 | 5887.3 | 1582.6 | 3707.1 | 6482.7 | 1.6 | 0.1 | 5.7 |
| ss906 | 8/20/2014 | A | 5 | 2137.0 | 6014.3 | 1701.1 | 3538.2 | 6001.6 | 1.8 | 0.1 |  |
| ss906 | 8/20/2014 | A | 10 | 2086.5 | 5862.2 | 1791.8 | 3540.0 | 5921.2 | 1.5 | 0.1 |  |
| ss901 | 8/20/2014 | A | 2 | 3601.5 | 5927.8 | 2977.4 | 5317.7 | 6879.9 | 1.0 | 0.1 | 8.2 |
| ss901 | 8/20/2014 | A | 5 | 3492.5 | 5912.2 | 2892.9 | 5310.2 | 6913.6 | 1.3 | 0.1 |  |
| ss901 | 8/20/2014 | A | 10 | 3586.2 | 5905.8 | 2926.8 | 5531.6 | 6970.8 | 1.2 | 0.1 |  |
|  |  |  |  |  |  |  |  |  |  |  |  |
